# RNA:DNA Ratio and Other Nucleic Acid Derived Indices in Marine Ecology

**DOI:** 10.3390/ijms9081453

**Published:** 2008-08-20

**Authors:** Maria Alexandra Chícharo, Luis Chícharo

**Affiliations:** Centre of Marine Sciences, CCMAR, University of Algarve. Campus de Gambelas, 8005-139 Faro, Portugal. E-Mail: lchichar@ualg.pt (L. C.)

**Keywords:** RNA:DNA ratio, ecological indicators, nucleic acid derived indices, marine ecology

## Abstract

Some of most used indicators in marine ecology are nucleic acid-derived indices. They can be divided by target levels in three groups: 1) at the organism level as ecophysiologic indicators, indicators such as RNA:DNA ratios, DNA:dry weight and RNA:protein, 2) at the population level, indicators such as growth rate, starvation incidence or fisheries impact indicators, and 3) at the community level, indicators such as trophic interactions, exergy indices and prey identification. The nucleic acids derived indices, especially RNA:DNA ratio, have been applied with success as indicators of nutritional condition, well been and growth in marine organisms. They are also useful as indicators of natural or anthropogenic impacts in marine population and communities, such as upwelling or dredge fisheries, respectively. They can help in understanding important issues of marine ecology such as trophic interactions in marine environment, fish and invertebrate recruitment failure and biodiversity changes, without laborious work of counting, measuring and identification of small marine organisms. Besides the objective of integrate nucleic acid derived indices across levels of organization, the paper will also include a general characterization of most used nucleic acid derived indices in marine ecology and also advantages and limitations of them. We can conclude that using indicators, such RNA:DNA ratios and other nucleic acids derived indices concomitantly with organism and ecosystems measures of responses to climate change (distribution, abundance, activity, metabolic rate, survival) will allow for the development of more rigorous and realistic predictions of the effects of anthropogenic climate change on marine systems.

## 1. Introduction

**Figure 1.**
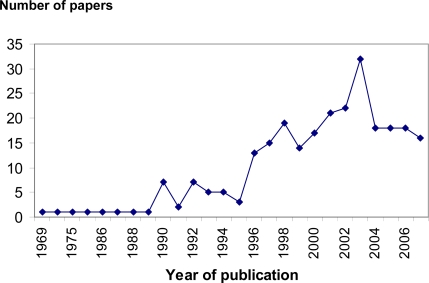
Evolution of the n° publications using RNA:DNA ratio and other major nucleic acid derived indices in marine ecology by year *. * source: http://www.scopus.com.scopeesprx.elsevier.com/

In marine ecology the determination of the *in situ* physiological state of marine organisms and communities are among of the main challenges. Therefore, the developments of new direct methods are necessary to gain a better understanding of physiology, trophic interactions and changes in composition and structure in aquatic ecosystems. Measurements of metabolic activity have been especially valuable as indicators of condition in studies of marine organisms, groups for which accurate determination of field metabolic rates is difficult [1]. Molecular methods based on nucleic acid derived indices [2] and the polymerase chain reaction has recently become an important tool in this field [3]. Many conceptually corrected biochemical measurement have been also proposed, but their implementation is often hindered by analytical complexities and problems in sampling, calibration and interpretation [4]. One of the most widely used nucleic acid derived indices in marine ecology is the RNA:DNA ratio. Since the RNA:DNA ratio was first proposed 38 years ago as a biochemical indicator of the physiological and nutritional state of aquatic organisms in natural environment [5] it has been continuously explored [6–10]. These indices have been applied with success in marine ecology in microbial communities’ [11] and in invertebrates and fishes [12–16]. All this information can be applied in the determination of the potential survival of a captured organism in the marine environment. With this knowledge prediction of target species’ population size can be made with special interest in marine conservation or marine fisheries studies of recruitment. It is widely recognized that high mortality during the larval stages occurs and may be due directly to starvation or to poor feeding conditions, which reduce larval growth rate and increase the duration of exposure to potential predators [17, 18]. Also RNA:DNA ratio have been used to test nutrient-productivity models by demonstrating tight linkages between nearshore oceanographic processes (such as upwelling) and benthic rocky intertidal ecosystems [1]. The search for reliable and accurate indices of nutritional condition and growth has been the focus of a steadily growing number of studies. Biochemical tools have been the object of most recent developments: they are mainly based on determination of nucleic acids indices. There was an increase number of published studies using nucleic derived indices, such as RNA:DNA ratio, particularly after 1990 (Figure 1).

This was due to much progress been made in improving the sensitivity of analytical methods, with the application of fluorometric techniques allowing measurement at the individual level, of small marine organisms such as marine larvae and copepods. In fact, these are among taxa where these indices have been more applied, especially the RNA:DNA ratio [12, 19, 20].

This paper characterizes the most used nucleic acid derived indices in marine ecology: 1) Organism level indices, such as RNA:DNA ratios, DNA/dry weight RNA/protein, 2) Population level as growth rate, starvation incidence and impact studies and 3) Community level such as trophic interactions, exergy indices and prey identification. The advantages and limitations of them will be also discussed.

## 2. Analysis of nucleic derived indices

### 2.1. Organism level indices: RNA:DNA ratio, DNA:dry weight and RNA:protein

To date the most widely-used biochemical index to determine marine organisms condition is the RNA:DNA ratio, which is an eco-physiological index of activity (growth, reproduction, secretion, etc.) under a given environmental condition [21]. This index gives a measure of the synthetic capacity of the cell and usually correlates with nutritional status [22]. The RNA:DNA ratio is based on the assumption that the amount of DNA, the primary carrier of genetic information, is stable under changing environmental situations within the somatic cells of a species [7], whereas the amount of RNA directly involved in protein synthesis, is known to vary with age, life-stage, organism size, disease-state and with changing environmental conditions [6]. Thus, organisms in good condition tend to have higher RNA:DNA ratios than do those in poor condition [7, 19]. In fact RNA:DNA ratios have been used on a wide range of marine organisms, mainly plankton, phytoplankton [11], zooplankton [13, 23, 24], and larval fish [7, 12, 25], but also juvenile and adult fish [6, 26], in bivalves [14, 27, 28], cephalopods [29, 30] and crustaceans [31, 32] and shown to reflect the nutritional status and may be useful in monitoring their physiological state in the field.

Some studies have advocated caution in the use of RNA:DNA ratios on the basis of techniques [27]. Nevertheless the Caldarone *et al.* study [33] of the intercalibration of four spectrofluorometric protocols for measuring RNA:DNA ratios in larval and juvenile fish showed that this problem can be solved if the ratio of the slopes of the standard curves are provided. This will facilitate intercomparability of RNA:DNA ratio results among laboratories using different spectrofluorometric methods. Moreover Berdalet [4, 10] proposed a detailed and rigorous protocol, with detection limit as lower as 1 ng for DNA, that can be used from microalgae to marine metazoan.

Others authors argue that the RNA:DNA ratios change with age in fishes [7, 34]. To avoid this size dependency problem it is advisable to use a residual based index from RNA content and an independently determined variable, such as standard length or dry weight for the removal of the allometric effect size (overall, simple regression of ln(RNA+l) on ln (Standard length)) [9, 35].

The analysis of the day variation for RNA:DNA, RNA residuals and RNA content detected in some fish larvae also shown that this aspect needed to be in consideration when sampling. In *Sardina pilchardus* Chicharo *et al.* [9] suggested that in twilight and early hours of the night the values of RNA:DNA were significantly higher. Rooker and Holt [34] also found diel changes in RNA/DNA ratios of red drum, *Sciaenops ocellatus*, larvae. This could result from an endogenous rhythm, i.e. circadian periodicities in endocrine activity induced by the light/dark regimen that raises the concentration of RNA in fish larvae during certain hours [9]. In fact the same diel patterns were observed for the bivalves *Ruditapes decussatus* and *Cerastoderma edule* [14] (Table 1). A solution to overcome the problem is to collect the organisms at the same period of day.

**Figure 2.**
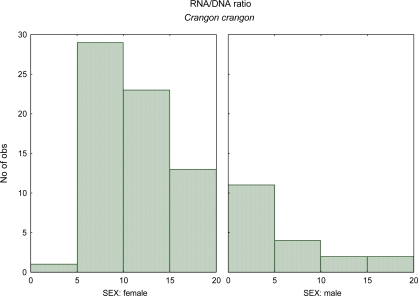
RNA:DNA ratio histogram of male versus female for the crustacean *Crangon crangon* (data from Chícharo *et al.* [31]).

The gender effect on the nucleic acids derived indices also is needed to be considered. The study of Chícharo *et al.* [31] aimed to quantify differences in RNA:DNA ratios and another indices based on nucleic acid concentrations between male and female fishes (*Pomatoschistus microps*), crustaceans (*Crangon crangon*) (Figure 2), and bivalves (*Ruditapes decussatus*).

There were significant differences in indices based on nucleic acid concentrations between males and females of all three species during the spawning season. RNA:DNA ratios were greater in females than in males, because of a greater content of RNA per unit dry weight in females and because of a greater content of DNA per unit dry weight in males. Sexual dimorphism and physiological and behavioural differences between males and females may explain these results. RNA:DNA ratios of adult organisms should be interpreted with caution because the effect of gender on nucleic acid concentrations may bias results if the sex ratio in the sample from which the results were derived is not representative of the sample. The RNA:DNA ratio may be underestimated if males are over-represented in the sample and overestimated if females are over-represented.

Special caution is also advised when selected selecting different kinds of tissue to determine the nucleic acids concentrations and ratios, since different tissues or body parts can have different RNA-or DNA-tissue relationships [37]. In some routine studies of larval condition based on RNA:DNA ratios, heads and/or guts are removed for further age and feeding analysis. Previous analyses are needed in order to determine the effect of this procedure in the results. Because previous major growth and starvation experiments in marine fish larvae described in literature to determine ratios and calibration responses of fish larvae were done analyzing the entire larvae, caution is needed when RNA:DNA ratios are obtained not using the entire organisms.

A high variability of RNA content, and thus RNA:DNA ratio, was revealed by estimates at the individual level and seems to be more related to feeding condition in late larval or juvenile stages than in yolk-sac and first-feeding larvae [2]. According to this author, an alternative is suggested, based on the DNA:DW, which appears more stable and sensitive to starvation during these early stages. The consistency of such a pattern is strongly supported by findings about regulation of ribosomal RNA content of tissues, and especially white muscle, by nutrition. Other nucleic acid derived indices, DNA:dry weight (DW) [2] and DNA:Carbon [38] are also sensitive to nutritional status, because cell weight is decreasing while DNA concentration is maintain constant during starvation. This DNA index increases when condition decrease, because more cells are present in the same weight of tissue. Reference values for good nutritional conditions, through DNA:DW are indicated in Table II. Higher values than the shown in this table indicate low fish larvae conditions. Nevertheless, there are also indications of size effects in the latter indices and that the response to nutritional condition is not sensitive as RNA:DNA ratio especially in bigger larvae [2].

### 2.2. Population indicators: growth rate, starvation incidence and fisheries impact

Over the past decades, RNA:DNA ratios have been used widely as an index of nutritional condition (see above section) and, to a lesser extent, to estimate growth rate. Nevertheless the earliest studies on the topic suggested that growth rates are related to RNA concentration in several species of copepods and other crustaceans [20, 23], and also in fish larvae [12]. The ratio of tissue RNA to DNA has proven to be a reliable estimator of recent growth of marine organisms. The amount of RNA in a cell varies in proportion to protein synthesis, whereas DNA concentrations remain fairly constant, even during starvation. Thus RNA:DNA ratio is an indicator of the protein-synthesizing potential of a cell [2, 7, 22].

**Figure 3.**
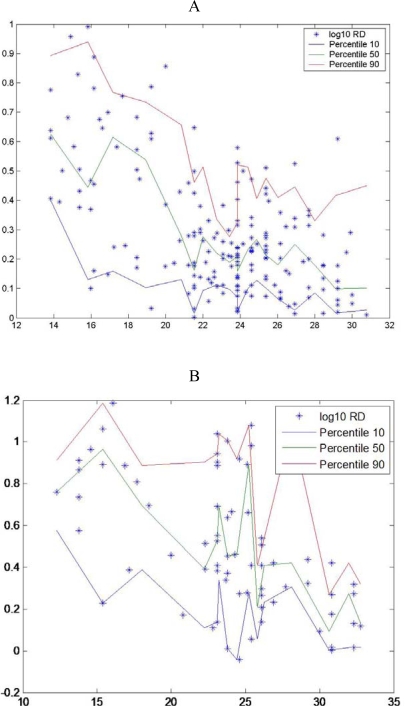

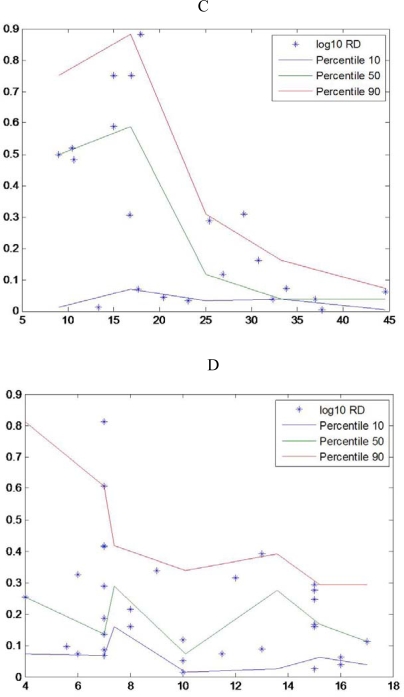
Observed nucleic acid ratio (y-RNA:DNA, log-transformed) in relation to standard larval (x) length (*) with the estimated 10^th^, 50^th^ and 90^th^ percentiles (thin solid lines) for A- *Sardina pilchardus* larvae, B- *Engraulis encrasicolus,* C-*Atherina presbyter* larvae and D- *Pomatoschistus s*pp larvae (data from [46]).

There are only a few widely-used approaches to estimating growth rates in marine organisms, among them are longitudinal sampling of a cohort, otolith microstructure analysis and RNA:DNA ratio. In the field, cohort analysis is most often a difficult task to accomplish and in the laboratory measurements of otolith microstructure, is a very laborious technique, but can provide offer a a detail history growth of each larva. Since Hovenkamp [39] demonstrated that growth rates from otoliths and nucleic acid ratios provide consistent measures of a larva’s state, we can infer that measuring an individual’s condition at one time provides an indication of its history. RNA:DNA ratio can provide estimates of growth rate over periods as short as one day and up to about one week [7]. This short time frame opens up the possibility of linking environmental conditions at the time of sampling to variability in growth and survival. If growth affected survival, survival rates would increase as time went on and vulnerable individuals, slow growing individuals were eliminated, as suggested in hypotheses about size-dependent mortality [40]. According to Pepin *et al.* [18] the smallest individuals have a condition distribution with low scatter and relatively close to maximum values. Nevertheless other authors found that the scatter decrease through development until metamorphosis, eg for sardine [41]. In different species, different relationships were found between dispersion of RNA:DNA ratios and length (Figure 3).

**Figure 4.**
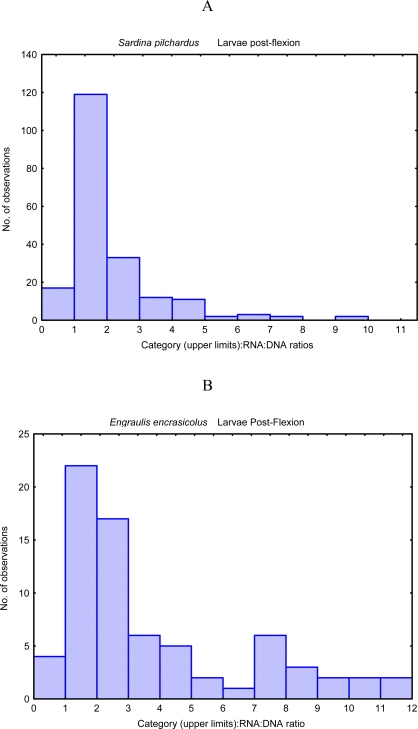

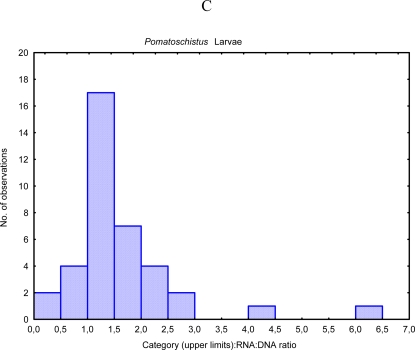
Histogram of RNA:DNA ratio of *Sardina pilchardus* (A), *Engraulis encrasicolus* (B) *Pomatoschistus* larvae(C), from North Atlantic (South Portugal coast) (data from [46]).

**Figure 5.**
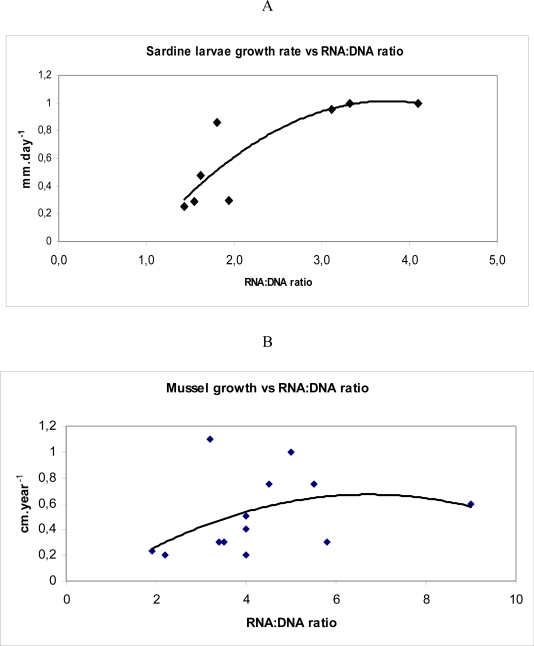
Relationship between A- larval cohorts growth rate and RNA:DNA ratio in *Sardina pilchardus* from North Atlantic (South Portugal coast) sampled with light trap (data from Chícharo unpublished), B – mussel annual growth rate and RNA:DNA ratio RNA/DNA of adductor muscle from the dominant mussel *Mytilus californianus* at 14 rocky intertidal sites in Oregon and California (data from [1]).

According to Pepin *et al.* [18] if survival probability (“fitness” measured through RNA:DNA ratio) is persistent, i.e. if it varies less during the larval life of one individual than it does among individuals, then in a population of larvae that starts with a distribution of fitnesses, with high values will form an ever larger fraction as time goes by. The most represented categories in an histogram of RNA:DNA ratios in different organisms are the intermediate classes (eg. Figure 4). This is due to the removal by predation and starvation of lower classes and because, in uppers categories, the probability of evasion of the organism to the capture device is high or because the maximum growth rate is not achieved with the highest values of RNA:DNA ratios [19]. In fact, this last hypothesis seemed to be more supported by the non-linear relationship between RNA:DNA ratio and growth rate described for adults mussels and sardine late larvae (15–30 mm) (Figure 5).

Besides the usually pattern of increasing RNA:DNA ratio with size is registered [7, 34, 42], eg. for early life stages of sardine [41] or the asymptotic value of RNA:DNA ratio, after obtaining a threshold larval size for anchovy (4–15 mm) [43]. By the opposite a pattern of decreasing RNA:DNA ratio with fish length was observed by [44, 45] for soleid juveniles and also for late larvae of clupeids [46]. The decrease of nucleic acid concentration during development was also indicated by Buckley *et al.* [22], Buckley and Bulow [47] and Mathers *et al.* [48]. According to these studies this is due to a switch from a higher proportion of hyperplasia in small fish to a higher fraction of hypertrophy in larger fish.

If for a certain life stage the relationship between RNA:DNA, temperature and growth is fixed, then if growth decreases with size and age it follows that RNA/DNA ratio would have to also decrease with size/age.

As late larvae decrease growth rates with age which is why it is very important not to directly compare growth rates or RNA:DNA ratio values of individuals with very different ages. A slower growing older larvae or juvenile may be in equally good condition to a faster growing younger larvae and a decrease in RNA:DNA ratio with age most likely reflects a decrease in growth rate but not necessarily a decrease in condition. The increased rate of chemical reactions at higher temperatures means that a higher growth rate can be achieved with the same or lower RNA concentration, and also that the RNA concentration associated with maximal growth rates declines with increasing temperature. Temperature is the dominant growth factor when larvae have adequate food supplies. Conversely, when the temperature range is narrow, food availability becomes the predominant factor for growth and condition [12].

In recent work, RNA:DNA ratios were also related to egg production rates of copepods [49]. RNA content or RNA:DNA ratios are also considered as indices of growth rates or reproductive capacity of marine copepods. Such an index would simplify the measurement of growth and egg production and would allow more extensive sampling in the field by eliminating the need for incubation of animals or successive sampling of cohorts.

Houlihan *et al.* [36] have questioned the use of the RNA:DNA index for calculation of instantaneous growth, since they found no correlation between this index and growth rate in the crab, *Carcinus maenas*. The authors argue that this is happens because protein deposition is dependent of protein synthesis but also of breakdown. Aditionally crustacean growth is also different from that of fishes. For those authors, RNA:protein is more adequate to detect changes in growth rate, because this index is more closely related to protein deposition. The RNA:protein is also very useful to detected starvation in fishes, since proteins forms an important energy source during long term food deprivation [50]. To assess starvation using RNA:DNA ratio it is necessary to determine the level below which the larvae will be classified as starving. The idea of the “critical ratio” was originally discussed by Robinson and Ware [19] and is based on a model of the general relationship between RNA:DNA ratio, temperature and protein growth rate determined and reported by Buckley [12]. Robinson and Ware [19] defined the “critical ratio” as the RNA:DNA ratio of an animal when larval protein growth rate is zero. They calculated the “critical ratio” for *Clupea harengus* using Buckley’s general model by setting protein growth rate to zero and solving for RNA:DNA ratio at the temperature which they captured the larvae. Rooker and Holt [34] suggested caution be used when applying this ratio to new species in field studies due to the inherent developmental variation seen in RNA:DNA ratios of many species of marine teleost larvae.

A solution to this problem is to calculate for each species, under controlled conditions, the mean RNA:DNA ratios of larvae deprived of food. This kind of calibration is usually done only with fed and starved laboratory reared larvae [12, 19, 28]. The results of such studies should be regarded with caution as laboratory conditions hardly simulate natural conditions [51]. Chícharo [52] assessed the RNA:DNA ratio indicative of starvation in *Sardina pilchardus* from a field experiment (ratios less than the value of 1.3). According to Clemmesen [7] there is a species-independent minimum RNA:DNA value around 1 necessary for survival. Nevertheless usually the critical values for evaluation of starvation published have used a length-and–age-independent RNA:DNA ratio, which did not account the probable variation this ratio with development.

The knowledge of the minimum values of RNA:DNA ratio needed for survival can also be useful in fisheries stress studies. Few investigations have focused on the influences of fishing methods on the health status of the target population. Nowadays there is a need to include the indirect mortality incurred during or post-escape from fishing gear. This issue is difficult to study, especially with organisms like fish that are very sensitive to handling. The condition indices, such as the nucleic acids derived indices referred to herein have been used to assess indirect mortality in bivalve dredge fisheries. Fishing by dredging is a source of acute and chronic stress for bivalve populations, especially to under-sized clams, which represent the population reservoir [53]. Usually, during the fishing process the juveniles are collected by fishing gear, tightly packed inside the fishing net, and some submitted to deck exposure, before being thrown back into the sea. In addition to this short-term physical impact, more intense in hydraulic dredges, all dredging activity also produce cumulative disturbances by successive mechanical impact and by the periodic sediment re-suspension. Knowledge of organism-level responses to induced stress is essential for understanding its adverse effects and the strategies adopted by organisms to tolerate such stress [54]. Then there is a need is to establish reliable indicators for tolerance, including the ability to recover. There is also a need in future studies to analyse this issue in other species, such as fishes and with other fishing gears, such as trawls. From modeling simulations the results indicate that improvement of trawl selectivity would have noticeable indirect effects on target demersal fish species [55].

Some of these stress indices use biochemical ratios or metabolic rates that integrate important part of life history of the individual. Chícharo and Chícharo [28] established that, among *Ruditapes decussatus* juveniles in Ria Formosa (Portugal), survival was not guaranteed when the RNA:DNA ratio was lower than 1. This value was, recently used for *Spisula solida* and *Donax trunculus* in a laboratory simulation of dredge stress, but after several hours of mechanical stress, this low value was never reached [56]. This agree with the fact that this indices can not be used as short-term or acute stress indicators, but they can be useful in detecting the well being of the bivalves under cumulative stress conditions, such as mechanical stress due to dredging activity, to spawning activity or to starvation conditions that follow excessive re-suspension of the sediment. There are important limitations on the use of this kind of index especially because to the difficulty in distinguished between natural versus dredging impact in their condition variation. Once again a well described seasonal and during the species life cycle is necessary in order to be able to separate both sources of index variations. The baseline dataset must cover natural variations and seasonal patterns in order to provide the context within which to determine if a change constitutes an impact. It is important to determine the thresholds of acceptability in any particular environment, in terms of the tolerance of the species present, and to relate this, for example, to the environmental change caused by the re-suspension or periodic mechanical impact, particularly the concentration of re-suspended sediment that can be tolerated over the background concentration. Such thresholds will be site specific and species specific. Therefore to correctly assess the impact of a fishing gear on a population it is necessary to establish the threshold level of the biochemical or physiological or behavioural indicator being used; i.e. the level below which the organism would be classified as severe stressed, which implies its minimal survival condition.

### 2.3. Community level: trophic interactions, biomass, prey identification and exergy indices

One of the most compelling applications of biochemical indices in ecological studies has been in testing models that predict changes in the structure of ecological communities—species distribution, species abundance, and the strength of interactions between competitors or between predators and prey—along environmental gradients in the rocky intertidal region. Two classes of models have been developed and tested using biochemical indicators of metabolism and stress: nutrient productivity models (N/P models) and environmental stress models or ESMs [57, 58]. These models assume that species interactions ultimately drive community structure but that communities are organized along environmental gradients, and severe habitats will have fewest number of species and lowest productivity, whereas the mildest habitats will have higher productivity, biomass, and species diversity. The two models are complementary: N/P models emphasize impacts of forces at the base of the food web on higher trophic levels (bottom-up effects), whereas ESMs assume that community structure is driven by variation in strength of interactions between basal species (algae, suspension feeding invertebrates) and foragers along environmental stress gradients (top-down effects). Both models have been important to a qualitative understanding of rocky intertidal community dynamics, and the application of biochemical indicators to these classical ecological problems has allowed for rigorous quantification of the relationships between environmental variation and community structure. Near shore primary productivity and RNA/DNA of adductor muscle from the dominant mussel at 14 rocky intertidal sites in Oregon and California. were described in the review of [1]. In this study, average summer (1999, 2000) chlorophyll-a concentrations in nearshore water were determined as RNA:DNA for mussels and nearshore primary productivity predicted mussel condition, as indexed by RNA:DNA (y = 2.68161 + 0.54788x; F1,11 = 5.0, p < 0.05). According to the author, driven by differences in nearshore primary productivity and by the delivery of high-quality phytoplankton during upwelling events, this results in radically different community structures, because the N/P variation is driving community structure, then increased energy input into suspension-feeder growth and reproduction increases their size and abundance, effectively increasing the carrying capacity of foraging predators by increasing their food supply, assuming space is not limiting the abundance of foragers. In fact regions with periods of high upwelling have more robust suspension-feeders and predators than regions of low upwelling.

The ability to obtain information about *in situ* physiological status for key communities in the sea, not only for benthic but also for plankton is vital for understanding the mechanisms structuring marine ecosystems. Zooplankton also plays a central role in the marine food web as selective predators and nutrient regenerators, and as mediators of energy to higher trophic levels [59]. Microzooplankton is generally the major consumers of phytoplankton in the sea, whereas mesozooplankton, dominate biomass of marine plankton and are key prey for higher trophic levels [60]. DNA concentrations may be a good measure of plankton living biomass and RNA:DNA ratios is also a good indicator of growth rates of microbial communities. Measuring biomass and growth rates in these planktonic organisms is a difficult analytical problem, but such knowledge is essential for answering fundamental questions in biological oceanography [11]. Measurements of RNA or DNA concentrations have not been utilized extensively, for investigating growth rates or biomass of microbial marine communities, as for large organism. One of the reason that can be cited is because hypothesized, that the presence of detrital nucleic acid in seawater [5]. Nonetheless presence of large amounts of extra- and intra-cellular RNAses and the rapid turnover of RNA in cells, suggests it is not very likely to be present in non-living organic matter [11]. The data and discussion presented by the last authors suggest that detrital DNA is not as much of a problem as originally hypothesized.

Mesozooplankton feed on a wide range of prey, and there are presently no methods available to directly quantify *in situ* zooplankton feeding on all different prey types. Therefore, the development of a new nonintrusive direct method is necessary to gain a better understanding of the trophic interactions in aquatic ecosystems. Molecular methods based on the polymerase chain reaction have recently become an important tool to study predation by arthropods, particularly insects [61]. According to Nejstgaard *et al.* [3] using a calanoid copepod as a model system, found that 18S ribosomal DNA originating from *E. huxleyi* was unambiguously detected in whole DNA extracts from copepods and from their fecal pellets. These results also suggest that prey DNA may be quantified for determination of prey-specific zooplankton feeding rates, and to trophic interactions between individual predators and all their prey in the complex natural plankton can be determined.

At a community level, other potential use of nucleic acids derived indices, such as DNA concentrations is for the empirical determination of exergy. In disturbed aquatic ecosystems a useful indicator of the anthropogenic impacts is the ecological exergy [62]. Ecological exergy was developed based on the exergy analysis in thermodynamics and the characteristics of living systems, that would consider both energy quantity and energy quality. This author suggested approximate calculations which could take into account the higher organization of some organisms and consequently its higher contribution to the exergy estimation. Based on the assumption of a common reference state (detritus or dead organic matter), Jørgensen [62] provided an approach for the approximated estimation of ecological exergy in terms of the probability (*Pi*) of producing organic matter (detritus) and the probability of ‘selecting’ its corresponding ‘genetic information’ (*Pi*,*a*), for each component, as follows:

$$Ex\;\approx \;-RT\sum_{i}^{N}Ci\;\text{ln}(Pi)$$

with *P_i_*=*P*_1_ · *P_i_,_a_* and *P_i,a_*=20^−700 *g*^, where *R* is the gas constant, *T* the absolute temperature, *c_i_* the concentration in the ecosystem of the component *i* and 700·*g* stands for an average value for the number of encoded amino-acids. With reasonable approximations, it can be computed as:

$$Ex/R\cdot T=\sum \beta_{i}c_{i}$$

where *ci* is the biomass concentration of species *i* and β*i* is a weighing factor expressing the ‘quantity of information’ embedded in the biomass [62]. This expression allows the computation of an ecological function associating to ecosystems its composition and biological structure (information), and it may be taken as an operative estimate for the ‘distance’ to a reference state assumed as a reference environment, where all components are inorganic and homogeneously distributed without gradients. Consequently, choosing detritus as a reference level (i.e. β1), the ‘genetic information’ content of organisms may be used to estimate β for different organisms (taking β as a discriminator of the organizational level of organisms relatively to detritus reference level). Therefore, it has been proposed to take into account the number of genes to determine the different exergy conversion factors (β). However, this proposal requires the knowledge of the total number of genes for many species, data not available for most species.

In the absence of these data, calculations are done based on rough estimates, obtained with flow cytometry [63]. The exergy index has been used with success when separating marine benthic ecosystem with different level of anthropogenic impacts [64]. This approach in the future can be also used in marine planktonic communities.

## 3. Conclusions

The nucleic acid derived indices reviewed in this study showed how useful they are in understanding structure and processes in marine ecosystems, without laborious work of counting, measuring and identification of small marine organisms. However, significantly more research under controlled laboratory and field conditions will be required in future. There is a need for baseline, species-specific validation studies before the generalized application of nucleic acid-based indices to ecological issues, such as ecophysiology of individuals, growth rate, fish and invertebrate recruitment failure, trophic interactions in marine environment, detecting anthropogenic impacts and natural changes. *In situ* calibration of indices minimum and maximum values under differing temperature regimes, and in depth analysis of how does their distribution change in response to variations in nutritional environmental condition or predation pressure, are also needed. Using indicators, such RNA:DNA ratios and other nucleic acids derived indices concomitantly with organism and ecosystems measures of responses to climate change (distribution, abundance, activity, metabolic rate, survival) will allow for the development of more realistic predictions of the effects of global change on marine systems.

## Figures and Tables

**Table 1.** Two way ANOVA results of RNA:DNA ratio in *Ruditapes decussatus* spat when the effects Night/Day (1) and Fed/Starved (2) were analyzed (results according to Chicharo *et al.* [14]).

Two way ANOVA	df effect^A^	MS effect^B^	Df error	F	p
1	1	35.076	99	4.42	0.03
2	1	79.952	99	10.09	0.001

A Table note: df-Degrees of freedom

B Table note: MS-Mean Square

**Table 2.** Relative DNA content (in ug.mg^−1^ dry weigth) (DNA/DW) measured in fed larval fish of different species.

Species	DNA:DW	Source
*Sardina pilchardus*	20–27	[41]
*Solea solea*	10–28	[2]
*Scophthalmus maximus*	10–20	[65]
